# Impacts of Freezing Temperature Based Thermal Conductivity on the Heat Transfer Gradient in Nanofluids: Applications for a Curved Riga Surface

**DOI:** 10.3390/molecules25092152

**Published:** 2020-05-05

**Authors:** Adnan, Syed Zulfiqar Ali Zaidi, Umar Khan, Naveed Ahmed, Syed Tauseef Mohyud-Din, Yu-Ming Chu, Ilyas Khan, Kottakkaran Sooppy Nisar

**Affiliations:** 1 Department of Mathematics, Mohi-ud-Din Islamic University, Nerian Sharif AJ&K, Trarkhel 12080, Pakistan; adnan_abbasi89@yahoo.com; 2 Department of Mathematics, COMSATS University Islamabad, Abbottabad Campus, Abbottabad 22010 Pakistan; zzaidi@cuiatd.edu.pk; 3 Department of Mathematics and Statistics, Hazara University, Mansehra 21120, Pakistan; umar_jadoon@hu.edu.pk or; 4 Department of Mathematics, Faculty of Sciences, HITEC University Taxila Cantt, Taxila 47070, Pakistan; nidojan@gmail.com; 5 University of Multan, Multan 60000, Pakistan; syedtauseefs@hotmail.com; 6 Department of Mathematics, Huzhou University, Huzhou 313000, China; chuyuming@zjhu.edu.cn or; 7 Faculty of Mathematics and Statistics, Ton Duc Thang University, Ho Chi Minh City 72915, Vietnam; 8 Department of Mathematics, College of Arts and Sciences, Prince Sattam bin Abdulaziz University, Wadi Aldawaser 11991, Saudi Arabia; n.sooppy@psau.edu.sa

**Keywords:** curved Riga surface, Al_2_O_3_ nanoparticles, thermal conductivity, freezing temperature, curvature, heat transfer

## Abstract

The flow of nanofluid over a curved Riga surface is a topic of interest in the field of fluid dynamics. A literature survey revealed that the impacts of freezing temperature and the diameter of nanoparticles on the heat transfer over a curved Riga surface have not been examined so far. Therefore, the flow of nanoparticles, which comprises the influences of freezing temperature and nanoparticle diameter in the energy equation, was modeled over a curved Riga surface. The model was reduced successfully in the nondimensional version by implementing the feasible similarity transformations and effective models of nanofluids. The coupled nonlinear model was then examined numerically and highlighted the impacts of various flow quantities in the flow regimes and heat transfer, with graphical aid. It was examined that nanofluid velocity dropped by increasing the flow parameters *γ* and *S*, and an abrupt decrement occurred at the surface of the Riga sheet. The boundary layer region enhances for larger *γ*. The temperature distribution was enhanced for a more magnetized nanofluid, and the thermal boundary layer increased with a larger *R* parameter. The volume fraction of the nanoparticles favors the effective density and dynamic viscosity of the nanofluids. A maximum amount of heat transfer at the surface was observed for a more magnetized nanofluid.

## 1. Introduction

Heat transfer investigation in nanofluids is a rich research direction in the field of fluid dynamics. Nanofluids have rich heat transfer characteristics in comparison with regular liquids. Therefore, in industries, the use of nanofluids in engineering and technological processes is preferable. For different industrial products, a huge amount of heat transfer required. Regular fluids like ethylene glycol, engine oil, kerosene oil and water fail to provide remarkable heat transfer amounts to accomplish the process of production. Due to high heat transfer characteristics, nanofluids are used instead of regular fluids. The roots of nanofluids are spread across the fields of electrical engineering, biotechnology, electronics and in computer chips.

The idea of heat transfer improvement in the regular liquids was raised in late 18th century. The researchers, scientists and engineers focused on such a significant idea which overcame the issues of industrialists and engineers. Finally, they added newly developed fluids in the list of fluids which are called nanofluids. Nanofluids are composed of a mixture of regular liquids and nano sized particles of various oxides and metals (γAl_2_O_3_, CuO, Ag, Cu, Fe_3_O_4_, MWCNTs, SWCNTs, Al_2_O_3_) which are thermally compatible. The regular liquids in nanofluids are known as base or host fluids. Due to high heat transfer characteristics, nanofluids became very popular among the researchers, scientists and engineers.

For the heat transfer enhancement in the nanofluids, thermal conductivities of the nanosized material and host liquid play the role of a backbone. Hamilton [1] proposed a thermal conductivity model, and nanoparticle shape factors emerged in the model. They considered three different shaped nanoparticles depending upon the shape factor. Koo and Kleinstreuer [2,3] introduced thermal conductivity correlations for ethylene glycol (EG) and oil saturated by CuO nanoparticles. Moreover, to enhance the heat transfer rate, they ingrained the temperature effects in the correlations. Thermal conductivity correlation for spherical shaped nanomaterials was introduced by Bruggemann [4]. They proposed the model at a high volume fraction of the nanoparticles. The extended thermal conductivity correlation considering *n* = 3 (shape factor) was introduced by Wasp [5]. A unique thermal conductivity correlation for H_2_O saturated by Al_2_O_3_ nanoparticles was discussed by Li and Peterson [6]. For better enhancement of heat transfer, they incorporated the influences of temperature and fraction factor into the model.

A thermal conductivity model which is reliable for oxides and metallic nanoscaled particles was discussed by Patel et al. [7] in 2010. They introduced the influences of the diameter of nanoparticles and temperature in the correlation. In 2010, Godson et al. [8] proposed a thermal conductivity model which is suitable for Ag/H_2_O composition. Thermal conductivity correlation for H_2_O saturated by Al_2_O_3_ nanoparticles was proposed by Corcione [9]. They considered the impacts of freezing temperature in the model and found fascinating results. Nanofluids became very popular among the engineers, scientists and industrialists. Researchers started to analyze the behavior of different thermal conductivity models on the heat transfer rate.

In 2019, Ahmed et al. [10] investigated the flow of magnetized nanofluid over a curved geometry. For mathematical analysis of the model, they utilized numerical techniques and presented the results for the flow regimes, heat transfer rate, and skin friction coefficient. Lately, Afridi et al. [11] reported the nanofluid flow over a curved surface, and for novelty of the analysis they incorporated the viscous dissipation phenomenon in the energy equation and examined significant variations in the temperature profile of the nanofluid. Ahmed et al. [12] reported the rotating flow of nanofluid squeezed between two parallel plates. They considered the composition of two fluids, namely water and ethylene glycol (EG) saturated by γAl_2_O_3_ nanoparticles. They presented fascinating results for the flow regimes and the local rate of heat transfer. The role of KKL thermal conductivity model in the heat transfer rate described by Sheikholeslami et al. [13] in 2016. Furthermore, they incorporated the magnetic field phenomenon in the momentum equation and observed interesting behaviors of the nanofluid’s velocity and temperature profiles. Abbas et al. [14] explored the flow of nanofluid over a curved Riga geometry. They made the study fascinating by incorporating the phenomenon of magnetic field in the momentum equation. A mathematical analysis of the model numerical scheme has been adopted, and results are reported for the flow regimes, nusselt number, and skin friction coefficient.

Nanofluids composed of host liquids and carbon nanotubes are very familiar among researchers. Xu [15] discussed the nanofluid model by considering carbon nanotubes. Ramzan et al. [16] reported a Bioconvection model together with entropy phenomena over a vertical cone. They modeled the governing equations by incorporating the carbon nanotubes’ effects. The results for density motile, velocity, mass and temperature are pictured and discussed comprehensively. Lu et al. [17] examined the nanofluid flow over a thin film composed of carbon nanotubes. The influences of Cattaneo Christov heat flux and entropy generation are also ingrained in the governing nanofluid model. The heat transfer analysis in the nanofluid composed of carbon nanotubes and host liquid between opening and narrowing channel reported by Khan et al. [18] in 2017. They found fascinating results for the flow field and heat transfer characteristics. Ahmed et al. [19] examined the heat transfer phenomenon by incorporating thermal radiation effects in the energy equation between Riga plates. For thermal enhancement, they used the Xue thermal conductivity model for carbon nanotubes. The flow of nanofluid over a curved surface is reported by Saba et al. [20] in 2018.

A literature survey reveals that the heat transfer investigation in H_2_O composed of carbon nanotubes and the influences of freezing temperature and nanoparticles diameter over a curved Riga surface have not been reported so far. This analysis presents the heat transfer phenomenon in the nanofluids by altering different flow quantities.

## 2. Physical Interpretation of the Results

### 2.1. The Velocity and Temperature Distribution

**Figure 1:**
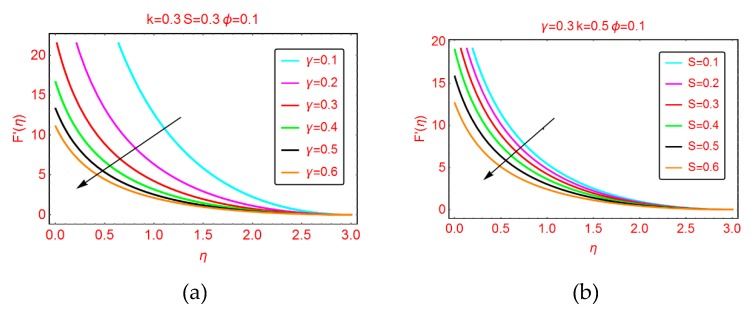
The velocity distribution for varying (**a**) *γ* and (**b**) *S*.

**Figure 2:**
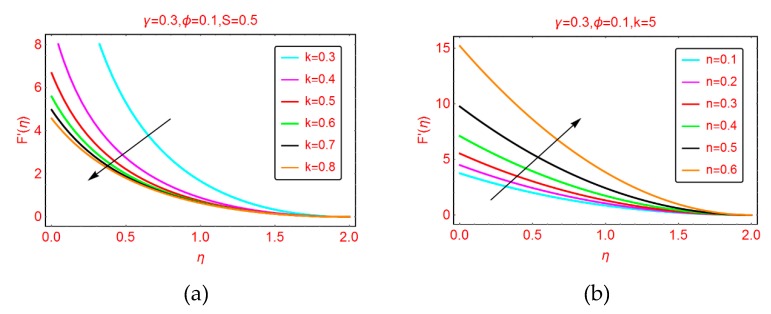
The velocity distribution for varying (**a**) *k* and (**b**) *n*.

**Figure 3:**
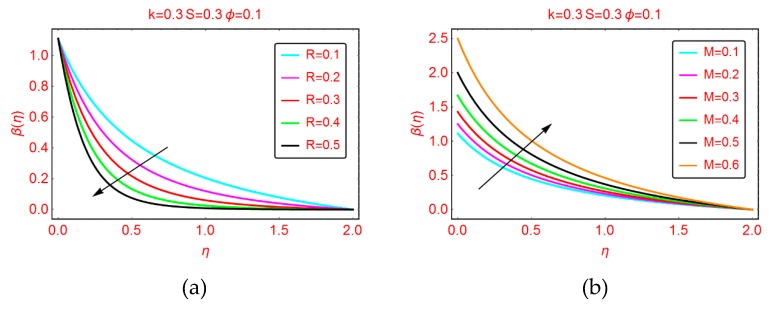
The temperature distribution for varying (**a**) *R* and (**b**) *M*.

This subsection highlights the behavior of nanofluid motion, F’(η) and thermal transport, β(η) for the parameters ingrained in the model. Figure 1, Figure 2 and Figure 3 are presented for this purpose.

#### 2.1.1. Effects of *γ* and *S* on F’(η)

Figure 1 shows the velocity behavior of the nanofluid for partial slip flow parameter *γ* and parameter *S*. It is perceived that the velocity F’(η) declines at the Riga surface abruptly for more slippery surfaces. Physically, the force of friction near the surface becomes stronger for the fluid particles adjacent to the Riga surface which resists the fluid particle motion. Therefore, the velocity F’(η) declines. Furthermore, the boundary layer thickness rises for more slippery surfaces. The behavior of F’(η) is very prominent in the locality of the Riga surface. These influences are shown in Figure 1a. The alterations in the nanofluid velocity F’(η) against growing values of the parameter *S* are shown in Figure 1b. From this, decrement in the nanofluid velocity F’(η) is examined at the surface. The boundary layer region starts beyond η > 2.5. Further, it was found that the velocity drops slowly for *S* in comparison with the velocity for *γ*.

#### 2.1.2. Effects of *k* and *n* on F’(η)

Figure 2a shows the behavior of nanofluid velocity against curvature of the Riga surface. It is perceived that larger curvature resists the nanofluid motion. The physical relevance of these effects is the increment in force of friction between the fluid particles and the Riga sheet. It is evident that by increasing the curvature of the sheet, the surface area increases, which causes more free space to be available for the flowing fluid. The fluid over the surface expanded and the force of friction enhances due to the larger surface, therefore, the nanofluid motion drops. The abrupt decreasing behavior of the nanofluid motion is perceived at the Riga surface because the force of friction is stronger in this region. On the other side, due to the larger curvature, the momentum boundary layer region increases. Figure 2b demonstrates the flowing behavior of the nanofluid for parameter *n*. The rapid increment in the nanofluid motion is examined near the surface for larger *n*. In this case, the boundary layer of the nanofluid decreases.

#### 2.1.3. Effects of *R* and *M* on β(η)

The thermal profile β(η) against stretching parameter *R* is shown in Figure 3a. The more stretched Riga surface opposes the temperature of the nanofluid. Physically, when the surface is stretched, the nanofluid motion drops. Due to the decrement in the fluid motion, collision between the fluid particles drops as a result of the temperature β(η) declining. Furthermore, it was found that for more stretched surfaces, the thermal boundary was enhanced. The behavior of nanofluid temperature β(η) against an induced magnetic field is shown in Figure 3b. The temperature β(η) increases the more magnetized the nanofluid. Near the region η = 0, an abrupt increment in the temperature β(η) is noticed. Further, more magnetized nanofluid resists the thermal boundary layer region in comparison with stretched parameter *R*.

### 2.2. Streamlines Profile

**Figure 4:**
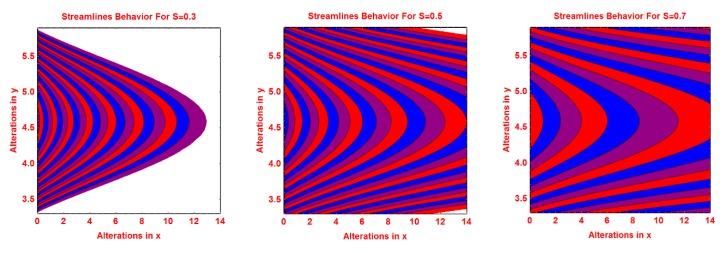
Effects of different values of *S* on the streamlines pattern.

**Figure 5:**
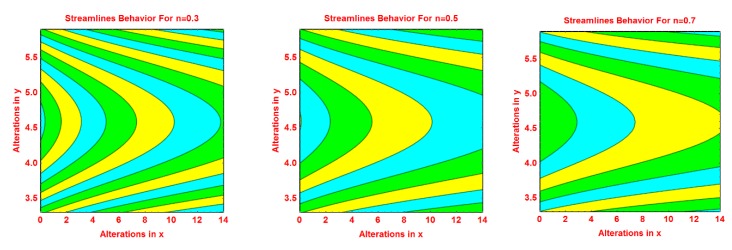
Effects of different values of *n* on the streamlines pattern.

**Figure 6:**
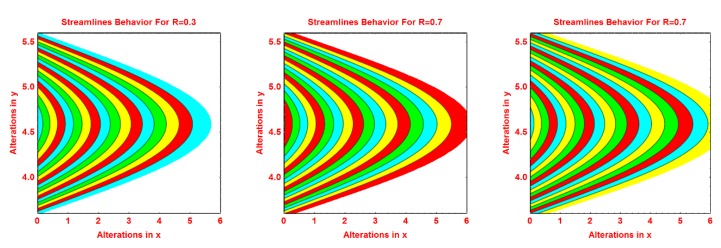
Effects of different values of *R* on the streamlines pattern.

**Figure 7:**
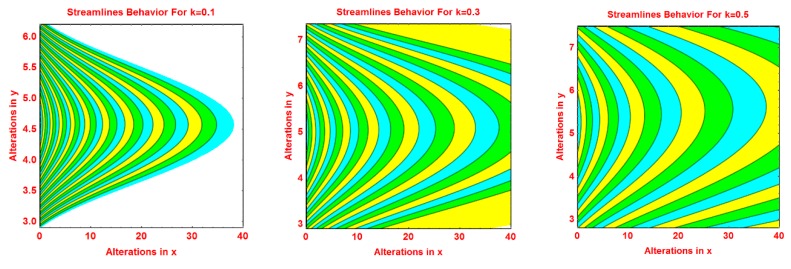
Effects of different values of *k* on the streamlines pattern.

The behavior of the streamlines pattern due to varying different flow parameters like *S*, *n*, *R* and curvature parameter *k* is presented graphically in this subsection. From Figure 4, it is clear that for smaller values of *S*, the flow pattern is more parabolic at the middle. As the values of *S* upturns, the flow pattern increases. The streamlined behavior is very fascinating for growing values of *n*. It can be seen that at the middle area of the pattern, the streamlines are more curved and of parabolic shaped and the curve expanded away from central curves. These effects are portrayed in Figure 5 for increasing values of *n*. Figure 6 and Figure 7 portray the pattern of streamlines for growing values of *R* and the curvature of the Riga sheet *k*, respectively.

### 2.3. Isotherms Profile

**Figure 8:**
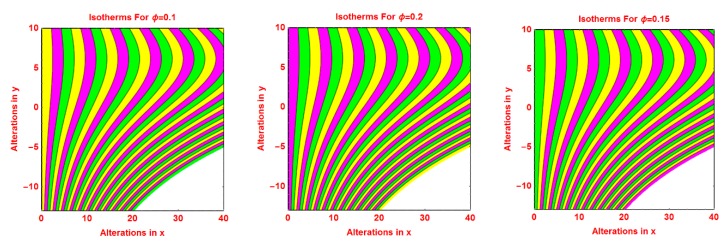
Effects of different values of *ϕ* on isotherms pattern.

**Figure 9:**
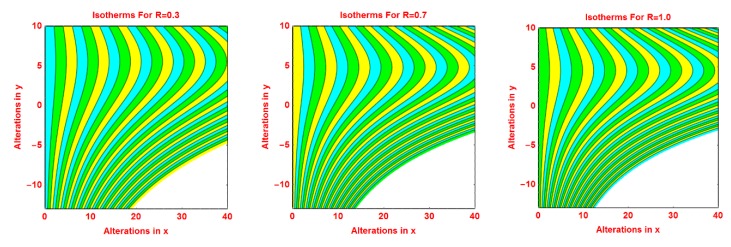
Effects of different values of *R* on isotherms pattern.

**Figure 10:**
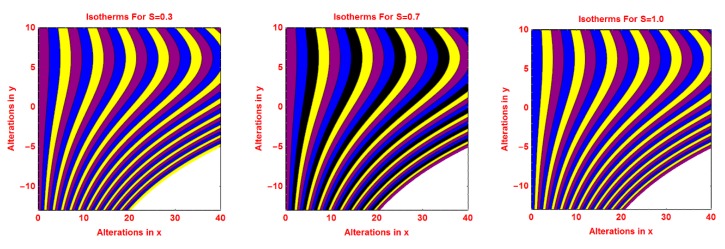
Effects of different values of *S* on isotherms pattern.

**Figure 11:**
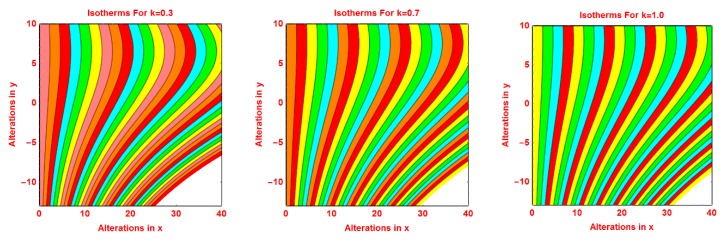
Effects of different values of *k* on isotherms pattern.

The pattern of isotherms for the volumetric fraction of nanoparticles *ϕ*, *R*, *S* and *γ* are presented in Figure 8, Figure 9, Figure 10 and Figure 11, respectively. It is investigated that the isotherms’ pattern is more curved for volume fraction *ϕ*, *R* and *S* parameters. These alterations in isotherms are examined in Figure 8, Figure 9 and Figure 10. The behavior of isotherms is fascinating for curvature parameter *k* and is depicted in Figure 11.

### 2.4. Thermophysical Characteristics

**Figure 12:**
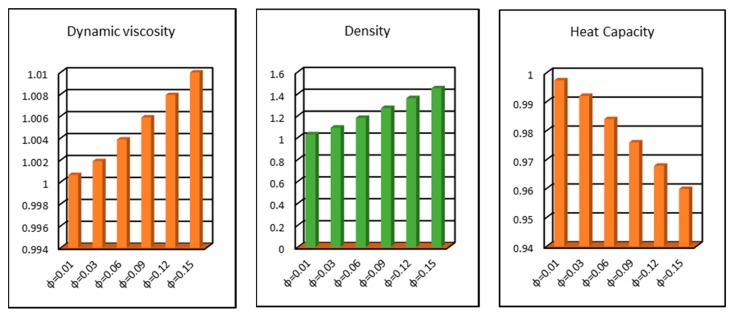
Effects of *ϕ* on effective dynamic viscosity, density and heat capacity.

The dynamic viscosity, effective density and heat capacity of the nanofluids is of great significance in the colloidal analysis. The fraction factor *ϕ* significantly alters the characteristics of these quantities. Figure 12 is shown for this purpose. The fraction factor domain is taken between 0–0.2. It is perceived that the effective characteristics of the nanofluids enhances by altering the fraction factor. The enhancement of these quantities significantly alters the flow characteristics such as the nanofluid motion, thermal transport, wall shear stresses and local heat transfer rate over the Riga surface.

### 2.5. Nusselt Number and Skin Friction

**Figure 13:**
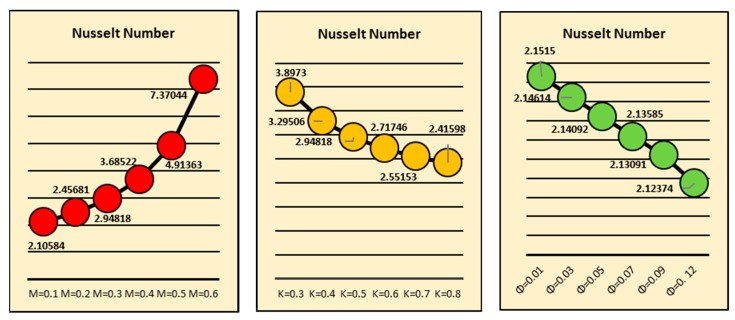
Effects of *M*, *k* and *ϕ* on the local Nusselt number.

**Figure 14:**
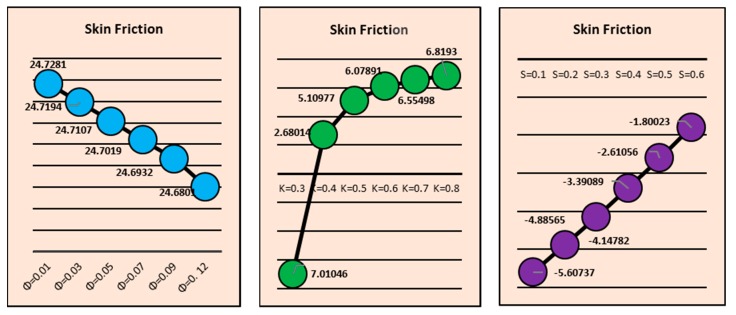
Effects of *ϕ*, *k* and *S* on the Skin friction coefficient.

Figure 13 reflects the transportation of the local heat transfer rate for the magnetic field, curvature and fraction factor at the Riga surface. It is perceived that there is more heat transport at the surface due to the stronger magnetic field. Physically, the induced magnetic field resists the fluid motion due to the fluid particles which come closer to each other and consequently, increase heat transfer at the surface. On the other side, larger curvature opposes the heat transportation. By increasing the surface curvature, the fluid particles scatter at the surface, which causes a decrement in fluid motion. Therefore, the rate of local heat transfer drops. Similarly, influences of fraction factor on the heat transfer rate are shown. The influences of fraction factor, curvature and *S,* on the shear stresses at the Riga surface are shown in Figure 14.

### 2.6. Reliability of the Study

Our nanofluid model is more general. However, under certain restrictions on the flow parameters, the results for −Re_s_C_F_ are compared with existing scientific literature. By setting *R_1_* = 1, *γ* = 0, *S* = 0, *ω* = 0, β* = 0, *θ* = 0, *ϕ* = 0, the following tabulated results are computed. From Table 1, it is perceived that our results are more reliable with the existing science literature, which proves the reliability of the presented analysis.

## 3. Materials and Methods

### 3.1. Model Formulation

#### 3.1.1. Statement and Geometry of the Model

**Figure 15:**
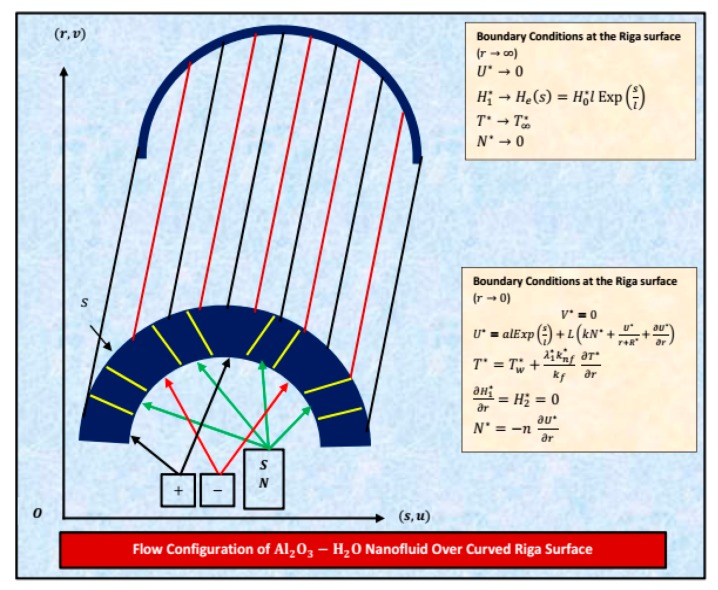
Flow of Al_2_O_3_–H_2_O over curved Riga surface.

The viscous incompressible flow of H_2_O by considering the freezing temperature and nanoparticles diameter effects over a curved Riga surface is under consideration. The Riga surface is placed in the curvilinear coordinates system. It is assumed that the host liquid and nanoparticles are thermally compatible. The Riga surface is capable of stretching. The curvilinear coordinates are denoted by *s* and *r,* and radius of the curve is taken as *r*. The flow of nanofluids over a curved Riga surface is shown in Figure 15.

#### 3.1.2. Governing Model and Similarity Transformations

The governing flow of nanofluid in light of aforementioned assumptions over a curved Riga surface is as follows [14]:

(1) $$\frac{\partial }{\partial r}\left(V^{*}\left(r+R^{*}\right)\right)+R^{*}\frac{\partial U^{*}}{\partial S}=0$$

(2) $$\frac{\partial }{\partial r}\left(H_{1}^{*}\left(r+R^{*}\right)\right)+R^{*}\frac{\partial H_{2}^{*}}{\partial S}=0$$

(3) $$\frac{U^{*}{}^{2}}{r+R^{*}}-\frac{1}{\rho_{nf}^{*}}\frac{\partial p^{*}}{\partial r}=0$$

(4) $$\begin{array}{c} V^{*}\frac{\partial U^{*}}{\partial r}+\frac{R^{*}U^{*}}{r+R}\frac{\partial U^{*}}{\partial S}+\frac{V^{*}U^{*}}{r+R^{*}}+\frac{1}{\rho_{nf}^{*}}\left(\frac{R}{r+R^{*}}\right)\frac{\partial p^{*}}{\partial s}=\left(\frac{\mu_{nf}^{*}}{\rho_{nf}^{*}}+\frac{k}{\rho_{nf}^{*}}\right)(\frac{\partial^{2}U^{*}}{\partial r^{2}}-\frac{U^{*}}{{\left(r+R^{*}\right)}^{2}}+\frac{1}{r+R^{*}}\;\frac{\partial U^{*}}{\partial r})-\\ \frac{K_{1}^{*}}{\rho_{nf}^{*}}\;\frac{\partial N^{*}}{\partial r}+\frac{\pi J_{0}^{*}M_{0}^{*}}{8\rho_{nf}^{*}}e^{-\frac{\pi }{a}r}+\frac{\mu_{e}^{*}}{4\pi \rho_{e}}\left(\left(\frac{R^{*}H_{1}^{*}}{r+R^{*}}\right)\frac{\partial H_{1}^{*}}{\partial s}+H_{2}^{*}\frac{\partial H_{1}^{*}}{\partial r}+\frac{H_{1}^{*}H_{2}^{*}}{r+R^{*}}\right) \end{array}$$

(5) $$\begin{array}{c} \left(\frac{R^{*}}{r+R^{*}}\right)\left(U^{*}\frac{\partial H_{1}^{*}}{\partial s}\;\right)+\frac{H_{1}^{*}H_{2}^{*}}{r+R^{*}}+V^{*}\frac{\partial H_{1}^{*}}{\partial r}-\left(\frac{R^{*}H_{1}^{*}}{r+R^{*}}\;\frac{\partial U^{*}}{\partial S}+\frac{V^{*}U^{*}}{r+R^{*}}+H_{2}^{*}\frac{\partial V^{*}}{\partial r}\right)\\ =\mu_{e}^{*}\left(\frac{\partial^{2}H_{1}^{*}}{\partial r^{2}}-\frac{H_{1}^{*}}{{\left(r+R^{*}\right)}^{2}}+\frac{1}{r+R^{*}}\frac{\partial H_{1}^{*}}{\partial r}\right) \end{array}$$

(6) $$V^{*}\frac{\partial N^{*}}{\partial r}+\frac{R^{*}U^{*}}{r+R^{*}}\frac{\partial N^{*}}{\partial s}=\frac{1}{\rho_{nf}^{*}}\left(\mu_{nf}^{*}+\frac{K_{1}^{*}}{2}\right)\left(\frac{1}{r+R^{*}}\frac{\partial N^{*}}{\partial r}+\frac{\partial^{2}N^{*}}{\partial r^{2}}\right)-\frac{K_{1}^{*}}{2\rho_{nf}^{*}}\left(\frac{\partial U^{*}}{\partial r}+2N^{*}+\frac{U^{*}}{r+R^{*}}\right)$$

(7) $$\frac{R^{*}U^{*}}{r+R^{*}}\;\frac{\partial T^{*}}{\partial s}+V^{*}\frac{\partial T^{*}}{\partial r}=\frac{k_{nf}^{*}}{{\left(\rho c_{p}\right)}_{nf}}\left(\frac{1}{r+R^{*}}\frac{\partial T^{*}}{\partial r}+\frac{\partial^{2}T^{*}}{\partial r^{2}}\right)$$

In the governing nanofluid model, $\rho_{nf}^{*}$, $k_{nf}^{*}$, $\mu_{nf}^{*}$ and (*ρc_p_*)*_nf_* are effective density, thermal conductivity, dynamic viscosity and specific heat capacitance, respectively. The conditions at the Riga surface and far from this, considering that the velocity slip and thermal jump are defined in the following way:

(8) $$\begin{array}{c} \mathrm{At}\;\mathrm{surface}\;r\to 0\\ V*=0\\ U^{*}=alExp\left(\frac{s}{l}\right)+L\left(kN^{*}+\frac{U^{*}}{r+R^{*}}+\frac{\partial U^{*}}{\partial r}\right)\\ T^{*}=T_{w}^{*}+\frac{\lambda_{1}^{*}k_{nf}^{*}}{k_{f}}\;\frac{\partial T^{*}}{\partial r}\\ \frac{\partial H_{1}^{*}}{\partial r}=H_{2}^{*}=0\\ N^{*}=-n\;\frac{\partial U^{*}}{\partial r}\\ \mathrm{Away}\;\mathrm{from}\;\mathrm{the}r\to \infty \\ U*\to 0\\ H_{1}^{*}\to H_{e}\left(s\right)=H_{0}^{*}l\mathrm{Exp}\left(\frac{s}{l}\right)\\ T*\to T_{\infty }^{*}\\ N*\to 0 \end{array}\}$$

In Equation (8), velocity slip is represented by *L*, thermal slip is $\lambda_{1}^{*}$, $T_{w}^{*}$ is the temperature at the Riga surface, $T_{\infty }^{*}$ denotes the temperature away from the Riga surface and *n* denotes the microgyration of micropolar nanofluid. The feasible invertible transformations under consideration for the nanofluid model are defined in the following manner:

(9) $$\begin{array}{c} T^{*}=T_{w}^{*}+\left(T_{\infty }-T_{w}\right)\beta \left(\eta \right)\\ \eta =\sqrt{\frac{a}{\nu_{f}}}r\\ U^{*}=alExp\left(\frac{s}{l}\right)F^{\prime }\left(\eta \right)\\ V^{*}=-\left(\frac{R^{*}}{r+R^{*}}\right)\frac{1}{l}\;\sqrt{a\nu_{f}}\mathrm{Exp}\left(\frac{s}{l}\right)F\left(\eta \right)\\ N^{*}=\sqrt{a\nu_{f}}\mathrm{Exp}\left(\frac{s}{l}\right)H\left(\eta \right)\\ P^{*}=\rho^{*}l^{2}\mathrm{Exp}\left(\frac{2s}{l}\right)p\left(\eta \right)\\ H_{1}^{*}=H_{0}^{*}l\mathrm{Exp}\left(\frac{s}{l}\right)G^{\prime }\left(\eta \right)\\ H_{2}^{*}=-H_{0}^{*}\left(\frac{R^{*}}{r+R^{*}}\right)Exp\left(s/l\right)\sqrt{\frac{\nu_{f}}{a}}\;G\left(\eta \right) \end{array}\}$$

#### 3.1.3. Effective Nanofluid Models

In order to improve thermal enhancement in the nanofluids, the following models are considered in this particular study [22]:

(10) $$\rho_{nf}^{*}=\left[\left(1-\phi \right)+\frac{\phi \rho_{p}}{\rho_{f}}\right]\rho_{f}$$

(11) $${\left(\rho C_{p}\right)}_{nf}=\left[\left(1-\phi \right)+\frac{\phi {\left(\rho C_{p}\right)}_{p}}{{\left(\rho C_{p}\right)}_{f}}\right]{\left(\rho C_{p}\right)}_{f}$$

(12) $$\mu_{nf}^{*}=\mu_{f}{\left(1-34.87{\left(\frac{d_{particle}}{d_{fluid}}\right)}^{-0.3}\phi^{1.03}\right)}^{-1}$$

(13) $$k_{nf}^{*}=k_{f}\left(1+4.4Re_{b}^{0.4}\overset{0.66}{\mathrm{Pr}}{\left(\frac{T}{T_{freezing}}\right)}^{10}{\left(\frac{k_{p}}{k_{f}}\right)}^{0.03}\phi^{0.66}\right)$$

In Equation (13), Reynolds number is *Re_b,_* due to the effects of brownian motion, and defined in the following formula:

(14) $$Re_{b}\left(\mu_{f}\right)=d_{p}\rho_{f}u_{b}$$

The velocity of brownian motion in Equation (14) calculated by the formula below:

(15) $$u_{b}=\frac{2Tk_{b}}{\left(\pi d_{p}^{2}\mu_{f}\right)}$$

In Equation (15), Stefan Boltzmann coefficient is denoted by *k_b_* and is equal to 1.380648 × 10^−23^ (*JK*^−1^) and the molecular diameter of the nanoparticles *d*_p_ is calculated by the following formula [23]:

(16) $$d_{f}=6M^{*}{\left(N^{*}\rho_{f}\pi \right)}^{-1}$$

The molecular weight of the host fluid and Avogadro number are represented by M* and N*, respectively. Further, the value of *d_f_* is calculated as

(17) $$d_{f}={\left(\frac{6\times 0.01801528}{998.62\times \left(6.022\times 10^{23}\right)\times \pi }\right)}^{\frac{1}{3}}=3.85\times 10^{-10}m$$

The thermophysical characteristics [22] of the tiny particles and host liquid given in Table 2:

#### 3.1.4. Nondimensional Nanofluid Model

After implementing the similarity transformations embedded in Equation (9), effective nanofluid models described in Equations (10)–(13), and appropriate partial derivatives in the governing model as described in Equations (1)–(7), with conditions at the surface of the Riga sheet and away from as embedded in Equation (8), the following nanofluid model comprising of the freezing temperature and the influence of nanoparticle diameter is attained:

(18) $$\begin{array}{c} \frac{1}{\left(1-\phi +\frac{\phi \rho_{s}}{\rho_{f}}\right)}\left(\frac{1}{1-34.87{\left(\frac{d_{particle}}{d_{fluid}}\right)}^{-0.3}\phi^{1.03}}+K_{1}^{*}\right)\left(F^{\prime \prime \prime \prime }+\frac{2F^{\prime \prime \prime }}{\left(k+\eta \right)}+\frac{F^{\prime }}{{\left(k+\eta \right)}^{3}}-\frac{F^{\prime \prime }}{{\left(k+\eta \right)}^{2}}\right)+\\ \frac{R_{1}k}{\left(k+\eta \right)}\left(FF^{\prime \prime }-F^{\prime \prime }F^{\prime }\right)+\frac{R_{1}}{{\left(k+\eta \right)}^{2}}\left(FF^{\prime \prime }-F^{\prime 2}\right)-\frac{R_{1}k}{{\left(k+\eta \right)}^{3}}FF^{\prime }-\frac{1}{\left(1-\phi +\frac{\phi \rho_{s}}{\rho_{f}}\right)}R_{1}K_{1}^{*}H^{\prime }-\\ \omega^{*}\text{Θ}\exp\left(-\omega^{*}\eta \right)+\frac{\beta^{*}}{\left(k+\eta \right)}\left(G^{\prime }G^{\prime \prime }+\frac{GG^{\prime }}{{\left(k+\eta \right)}^{2}}-GG^{\prime \prime }-\frac{G^{\prime }G^{\prime }+GG^{\prime \prime }}{\left(k+\eta \right)}\right)=0 \end{array}$$

(19) $$\begin{array}{c} \lambda^{*}\left(G^{\prime \prime \prime }+\frac{G^{\prime \prime }}{\left(k+\eta \right)}-\frac{G^{\prime }}{{\left(k+\eta \right)}^{2}}\right)+R_{1}(\frac{k}{\left(k+\eta \right)}G^{\prime }F^{\prime }-\frac{k^{2}}{{\left(k+\eta \right)}^{3}}\;GF+\frac{k^{2}}{{\left(k+\eta \right)}^{2}}GF^{\prime }-\frac{k}{{\left(k+\eta \right)}^{2}}FF^{\prime }-\\ R_{1}\left(\frac{k}{\left(k+\eta \right)}G^{\prime }F^{\prime }-\frac{k}{{\left(k+\eta \right)}^{2}}GG^{\prime }-\frac{k}{\left(k+\eta \right)}FG\prime \prime \right))=0 \end{array}$$

(20) $$\begin{array}{c} \frac{1}{\left(1-\phi +\frac{\phi \rho_{s}}{\rho_{f}}\right)}\left(\frac{1}{1-34.87{\left(\frac{d_{particle}}{d_{fluid}}\right)}^{-0.3}\phi^{1.03}}+\frac{K_{1}^{*}}{2}\right)\left(H^{\prime \prime }+\frac{H^{\prime }}{\left(k+\eta \right)}\right)-\frac{R_{1}}{2}\frac{K_{1}^{*}}{\left(k+\eta \right)}F^{\prime }H+\frac{R_{1}}{2}\frac{K_{1}^{*}}{\left(k+\eta \right)}H^{\prime }F-\\ \frac{1}{\left(1-\phi +\frac{\phi \rho_{s}}{\rho_{f}}\right)}\frac{\;\;R_{1}K_{1}^{*}}{2}\left(2H+F^{\prime \prime }+\frac{F^{\prime }}{\left(k+\eta \right)}\right)\;=0 \end{array}$$

(21) $$\frac{\left(1+4.4Re_{b}^{0.4}\overset{0.66}{\mathrm{Pr}}{\left(\frac{T}{T_{freezing}}\right)}^{10}{\left(\frac{k_{p}}{k_{f}}\right)}^{0.03}\phi^{0.66}\right)}{\left(1-\phi \right)+\frac{\phi {\left(\rho c_{p}\right)}_{s}}{{\left(\rho c_{p}\right)}_{f}}}\left(\beta^{\prime \prime }+\frac{1}{\left(k+\eta \right)}\beta^{\prime }\right)+\frac{kR_{1}}{\left(k+\eta \right)}\beta^{\prime }F-\frac{kR_{1}}{\left(k+\eta \right)}\beta =0$$

The dimensional boundary conditions reduced into the following dimensionless form:

(22) $$\begin{array}{c} \mathrm{At}\;\eta =0\\ F^{\prime }\left(\eta \right)=1+\gamma \left(\frac{1}{k}F^{\prime }\left(\eta \right)+F^{\prime \prime }\left(\eta \right)\left(1-n\right)\right)\\ F\left(\eta \right)=S\\ H\left(\eta \right)=F^{\prime \prime }\left(\eta \right)n\\ G\left(\eta \right)=0\\ G^{\prime \prime }\left(\eta \right)=0\\ \beta \left(\eta \right)=1+\frac{M\left(1+4.4Re_{b}^{0.4}\overset{0.66}{\mathrm{Pr}}{\left(\frac{T}{T_{freezing}}\right)}^{10}{\left(\frac{k_{p}}{k_{f}}\right)}^{0.03}\phi^{0.66}\right)}{k_{f}}\beta \left(\eta \right)\\ \mathrm{At}\;\eta \to \infty \\ F^{\prime }\left(\eta \right)=0\\ F^{\prime \prime }\left(\eta \right)=0\\ H\left(\eta \right)=0\\ G^{\prime }\left(\eta \right)=1\\ \beta \left(\eta \right)=0 \end{array}\}$$

In Equations (18)–(21), $K_{1}^{*}$ represents the microgyration number, *ϕ* is the fraction factor, *θ* is the modified Hartmann number, ω* is a dimensionless quantity, *k* is the dimensionless surface curvature, λ* is the reciprocal magnetic Prandtl number, *Pr* is the Prandtl number, *M* is the thermal slip parameter, *γ* denotes the partial slip parameter, and *R_1_* is the stretching parameter.

#### 3.1.5. Significant Quantities from Engineering Aspects

Quantities like skin friction coefficient and local nusselt number are very popular among the researchers and engineers. The dimensional formulas for the aforementioned quantities are as follows:

(23) $$C_{F}=\frac{\tau_{rs}}{\rho_{nf}^{*}u_{w}^{2}}$$

(24) $$N_{u}=\frac{q_{w}s}{k_{nf}\left(T_{w}^{*}-T_{\infty }^{*}\right)}$$

The shear stresses and heat flux are defined as

(25) $$\tau_{rs}=\frac{1}{\left(1-\phi +\frac{\phi \rho_{s}}{\rho_{f}}\right)}\left(\frac{1}{1-34.87{\left(\frac{d_{particle}}{d_{fluid}}\right)}^{-0.3}\phi^{1.03}}+K_{1}^{*}\right)\left(\frac{\partial u}{\partial r\;}+\frac{u}{R+r}+kN^{*}\right){↓}_{r=0}$$

(26) $$q_{w}=-\left(1+4.4Re_{b}^{0.4}\overset{0.66}{\mathrm{Pr}}{\left(\frac{T}{T_{freezing}}\right)}^{10}{\left(\frac{k_{p}}{k_{f}}\right)}^{0.03}\phi^{0.66})\right)\left(\frac{\partial T^{*}}{\partial r\;}\right){↓}_{r=0}$$

By incorporating the shear stresses and heat flux described in Equations (25), (26) in Equations (23), (24), the following self-similar forms for skin friction and local nusselt number are obtained:

(27) $$\sqrt{Re_{s}}\;C_{F}=\frac{1}{\left(1-\phi +\frac{\phi \rho_{s}}{\rho_{f}}\right)}\left(\frac{1}{1-34.87{\left(\frac{d_{particle}}{d_{fluid}}\right)}^{-0.3}\phi^{1.03}}+K_{1}^{*}\right)\left(F^{\prime \prime }\left(\eta \right)+\frac{F^{\prime }\left(\eta \right)}{k}-nK_{1}^{*}F^{\prime \prime }\left(\eta \right)\right){↓}_{\eta =0}$$

(28) $$\sqrt{Re_{s}}\;Nu=-\left(1+4.4Re_{b}^{0.4}\overset{0.66}{\mathrm{Pr}}{\left(\frac{T}{T_{freezing}}\right)}^{10}{\left(\frac{k_{p}}{k_{f}}\right)}^{0.03}\phi^{0.66})\right)\beta^{\prime }\left(\eta \right){↓}_{\eta =0}$$

where $Re_{s}=\frac{aU_{w}^{*}}{\nu_{f}}$ and is known as local Reynold number.

### 3.2. Mathematical Analysis

After further consideration, the nanofluid model comprising the set of Equations (18)–(21) is coupled with a system of a nonlinear nature. The closed form solutions are inconvenient for such a model. Therefore, a numerical approach to tackle the model is best. Thus, the Runge–Kutta scheme and the shooting method [24,25] are implemented for the solution purpose. For this, a set of ordinary differential equations with feasible conditions are required. The system of higher order ordinary differential equations (ODEs) is transformed into a system of first order ODEs via following transformations:

(29) $$\begin{array}{c} b_{1}^{*}=F,\;b_{2}^{*}=F^{\prime },\;b_{3}^{*}=F^{\prime \prime },\;b_{4}^{*}=F^{\prime \prime \prime }\\ b_{5}^{*}=G,\;b_{6}^{*}=G^{\prime },\;b_{7}^{*}=G^{\prime \prime }\\ b_{8}^{*}=H,\;b_{9}^{*}=H^{\prime }\\ b_{10}^{*}=\beta ,\;b_{11}^{*}=\beta \prime \end{array}\}$$

From Equation (29), we obtained:

(30) $$\begin{array}{c} b_{1}^{*}\prime =F\prime ,\;b_{2}^{*}\prime ={F^{\prime }}^{\prime },\;b_{3}^{*}\prime ={F^{\prime }}^{\prime \prime },\;b_{4}^{*}\prime =F\prime \prime \prime \prime \\ b_{5}^{*}\prime =G\prime ,\;b_{6}^{*}\prime ={G^{\prime }}^{\prime },\;b_{7}^{*}\prime =G^{\prime \prime \prime }\\ b_{8}^{*}\prime =H\prime ,\;b_{9}^{*}\prime =H\prime^{\prime }\\ b_{10}^{*}\prime =\beta \prime ,\;b_{11}^{*}\prime =\beta \prime \prime \end{array}\}$$

Plugging the values from Equation (29) in Equation (30), we reached the following:

(31) $$\begin{array}{c} b_{1}^{*}{}^{\prime }=b_{2}^{*},\;b_{2}^{*}{}^{\prime }=b_{3}^{*},\;b_{3}^{*}{}^{\prime }=b_{4}^{*},\;\;b_{4}^{*}\prime =F\prime \prime \prime \prime \\ b_{5}^{*}{}^{\prime }=b_{6}^{*},\;b_{6}^{*}{}^{\prime }=b_{7}^{*},\;b_{7}^{*}\prime =G^{\prime \prime \prime }\\ b_{8}^{*}{}^{\prime }=b_{9}^{*},\;b_{9}^{*}\prime =H\prime^{\prime }\\ b_{10}^{*}{}^{\prime }=b_{11}^{*},\;b_{11}^{*}\prime =\beta \prime \prime \end{array}\}$$

After incorporating these transformations into the model given in Equations (18)–(21) and supporting boundary conditions given in Equation (22), a system of first order initial value problems is attained. The accuracy of the scheme is fixed at 10^−6^ and the process of calculation is repeated unless the desired accuracy is obtained.

## 4. Conclusions

The colloidal analysis of Al_2_O_3_-H_2_O over a magnetized curved Riga surface is presented. The results for multiple flow parameters are shown over the region of interest. From the study, it is perceived that

- The flowing region increases for larger curvatures of the Riga surface which resists the nanofluid motion.
- The momentum boundary layer region increases for stretched Riga surface.
- The thermal behavior β(η) enhances near the Riga surface abruptly for more magnetized colloidal mixtures of Al_2_O_3_-H_2_O.
- More heat transportation at the Riga surface is perceived for a stronger induced magnetic field.
- The wall shear stresses decline for larger values of parameter *S*.
- Al_2_O_3_-H_2_O nanofluids are better to use for industrial uses regarding thermal transportation in the occurrence of magnetic field.

## Figures and Tables

**Table 1:** Reliability of the Study.

*k*	Sajid et al. [21]	Present
5	0.75763	0.757631
10	0.87349	0.873489
20	0.93561	0.93561
30	0.95686	0.970198
40	0.96759	0.942587
50	0.97405	0.974049

**Table 2:** Thermophysical Characteristics T = 310 K [22].

Properties	*d*_p_ (nm)	ρ (kg/ m^3^)	β (1/k)	*C*_p_ (J/kg K)	μ_f_ (kg/ms)	k (W/mk)	σ (S/m)
H_2_O	0.385	993	36.2 × 10^5^	4178	695 × 10^6^	0.628	0.005
Al_2_O_3_	33	3970	0.85 × 10^5^	765	---------	40	0.05 × 10^6^

## Nomenclature

*U**	the velocity along s direction
*V**	the velocity along r direction
*p**	pressure
$\mu_{nf}^{*}$	dynamic viscosity
$\rho_{nf}^{*}$	effective density
$k_{nf}^{*}$	thermal conductivity of the colloidal suspension
(*ρc_p_*)_*nf*_	heat capacitance of the colloidal suspension
*nf*	stands for nanofluid
$\lambda_{1}^{*}$	slip parameter
*ρ_p_*	particles density
*ρ_f_*	host fluid density
*μ_f_*	dynamic viscosity of host liquid
*k_p_*	tiny particles thermal conductivity
*k_f_*	host liquid thermal conductivity
*d_fluids_*	molecular diameter
*d_particles_*	tiny particles diameter
*ϕ*	volume fraction factor
*k_b_*	Stefan Boltzmann constant
$K_{1}^{*}$	micropolar parameter
*n*	microgyration parameter
*M*	thermal slip number
*γ*	partial slip number
*θ*	modified Hartmann number
*ω**	nondimensional parameter
*λ**	reciprocal magnetic Prandtl number
η	similarity variable
F’(η)	dimensionless velocity
β(η)	dimensionless temperature
*Re_s_*	local Reynolds number
*M**	molecular weight
*N**	Avogadro number
*Pr*	Prandtl number

## References

[1] Hamilton H.L., Crosser O.K. (1962). Thermal Conductivity of Heterogeneous Two-Component Systems. Ind. Eng. Chem. Fund..

[2] Koo J., Kleinstreuer C. (2004). A new thermal conductivity model for nanofluids. J. Nano. Res..

[3] Koo J., Kleinstreuer C. (2005). Laminar nanofluid flow in micro-heat sinks. Int. J. Heat Mass. Trans..

[4] Bruggeman D.A.G. (1935). Berechnung verschiedener physikalischer konstanten von heterogenen substanzen. I. Dielektrizitatskonstanten und leitfahigkeiten der mischkorper aus isotropen substanzen. Ann. Der Phy. Le..

[5] Wasp E.J., Kenny J.P., Gandhi R.L. (1977). Solid–Liquid Flow Slurry Pipeline Transportation.

[6] Li C.H., Peterson G.P. (2006). Experimental investigation of temperature and volume fraction variations on the effective thermal conductivity of nanoparticle suspensions (nanofluids). J. Appl. Phy..

[7] Patel H.E., Sundararajan T., Das S.K. (2010). An experimental investigation into the thermal conductivity enhancement in oxide and metallic nanofluids. J. Nano Res..

[8] Godson R.L., Mohan L.B., Wongwises D.S. (2010). Experimental investigation on the thermal conductivity and viscosity of silver—deionized water nanofluid. Exp. Heat Trans..

[9] Corcione M. (2011). Rayleigh–Be’nard convection heat transfer in nanoparticle suspensions. Int. J. Heat. Fluid. Flow.

[10] Ahmed Z., Al-Qahtani A., Nadeem S., Saleem S. (2019). Computational Study of MHD Nanofluid Flow Possessing Micro-Rotational Inertia over a Curved Surface with Variable Thermophysical Properties. Processes.

[11] Afridi M.I., Qasim M., Wakif A., Hussanan A. (2019). Second Law Analysis of Dissipative Nanofluid Flow over a Curved Surface in the Presence of Lorentz Force: Utilization of the Chebyshev–Gauss–Lobatto Spectral Method. Nanomaterials.

[12] Ahmed N., Khan U., Mohyud-Din S.T. (2017). Influence of an Effective Prandtl number Model on Squeezed Flow of γAl_2_O_3_-H_2_O and γAl_2_O_3_-C_2_H_6_O_2_ Nanofluids. J. Mol. Liq..

[13] Sheikholeslami M., Zia Q.M.Z., Ellahi R. (2016). Influence of Induced Magnetic Field on Free Convection of Nanofluid Considering Koo-Kleinstreuer-Li (KKL) Correlation. Appl. Sci..

[14] Abbas N., Malik Y.M., Nadeem S. (2020). Transportation of Magnetized Micropolar Hybrid Nanomaterial Fluid Flow over a Riga Curface Surface. Comp. Meth. Prog. Biomed..

[15] Xue Q.Z. (2005). Model for Thermal conductivity of carbon nanotubes based composites. Nanotech..

[16] Ramzan M., Mohammad M., Howari F. (2019). Magnetized suspended carbon nanotubes based nanofluid flow with bio-convection and entropy generation past a vertical cone. Sci. Rep..

[17] Lu D., Ramzan M., Mohammad M., Howari F., Chung J.D. (2019). A Thin Film Flow of Nanofluid Comprising Carbon Nanotubes Influenced by Cattaneo-Christov Heat Flux and Entropy Generation. Coatings.

[18] Khan U., Ahmed N., Mohyud-Din S.T. (2017). Heat transfer effects on carbon nanotubes suspended nanofluid flow in a channel with non-parallel walls under the effect of velocity slip boundary condition: A numerical study. Neu. Comp. App..

[19] Ahmed N., Khan U., Mohyud-Din S.T. (2017). Influence of Thermal Radiation and viscous dissipation on squeezed flow of water between two Riga plates saturated with carbon nanotubes. Coll. Surf. A-Physcio. Eng. Asp..

[20] Saba F., Ahmed N., Hussain S., Khan U., Mohyud-Din S.T., Darus M. (2018). Thermal Analysis of Nanofluid Flow over a Curved Stretching Surface Suspended by Carbon Nanotubes with Internal Heat Generation. App. Sci..

[21] Sajid M., Ali N., Javed T., Abbas Z. (2010). Stretching a Curved Surface in a Viscous Fluid. Chin. Phys. Lett..

[22] Alsabery A.I., Sheremet M.A., Chamkha A.J., Hashim I. (2018). MHD convective heat transfer in a discretely heated square cavity with conductive inner block using two-phase nanofuid model. Sci. Rep..

[23] Corcione M. (2011). Empirical correlating equations for predicting the effective thermal conductivity and dynamic viscosity of nanofluids. En. Conv. Mang..

[24] Khan U., Ahmed N., Mohyud-Din S.T. (2017). 3D Squeezed Flow of γAl_2_O_3_-H_2_O and γAl_2_O_3_-C_2_H_6_O_2_ Nanofluids: A Numerical Study. Int. J. Hydrogen. Energy..

[25] Ahmed N., Khan U., Mohyud-Din S.T. (2017). Unsteady Radiative Flow of Chemically reacting Fluid over a Convectively Heated Stretchable Surface with Cross-Diffusion Gradients. Int. J. Sci..

